# Evaluation of small grain cover crops as a sustainable nematode management strategy for *Meloidogyne incognita* and *Rotylenchulus reniformis* in the Southeastern U.S.

**DOI:** 10.2478/jofnem-2025-0021

**Published:** 2025-06-21

**Authors:** Sloane McPeak, Kara Gordon, Bisho Lawaju, Kathy Lawrence

**Affiliations:** 559 Devall Dr. CASIC Building, Auburn Univ, AL 36849

**Keywords:** Integrated Nematode Management, *Meloidogyne*, root-knot nematode, *Rotylenchulus reniformis*, reniform nematode, small grain cover crops

## Abstract

This experiment investigates five small grain winter cover crops including multiple genotypes of barley (*Hordeum vulgare* L), oats (*Avena sativa* L.), rye (*Secale cereale* L.), triticale (x *Triticosecale* Wittmack), and wheat (*Triticum aestivum* L.) as a sustainable nematode management strategy for *Meloidogyne incognita* (root-knot nematode) and *Rotylenchulus reniformis* (reniform nematode) in cotton production in a Southeastern U.S. Greenhouse (2019), and field experiments (2019–2021) evaluated these crops for nematode host status, forage quality, and grain yield.

Greenhouse experiments showed that all small grains had higher average *M. incognita* egg counts than a standard corn (Zea mays L.) variety. Overall, barley and wheat were suitable hosts (Rf>2), triticale and oat were moderate hosts (Rf=1-2), while three cultivars (Forerunner’ and ‘OG170039’ triticale, “ORO 4372’ oat) were poor hosts (Rf<1). In field trials, oat had the highest biomass and grain yield, followed by triticale, barley, rye, and wheat. Barley supported the highest population density of *M. incognita*. Oat, barley, and rye displayed similar population density of *R. reniformis* and were greater than triticale and wheat. Forage quality experiments showed oat with the highest biomass, wheat with the highest crude protein, and rye and triticale leading in fiber content. Oats had the greatest total digestible nutrients (TDN) and relative feed value (RFV), indicating superior digestibility. All small grains demonstrated high forage quality (RFV>100). Cover crop selection should be based on specific management and agronomic goals as nematode populations varied by cover crop but were low in all field trials. Further research on crop-specific responses and long-term effects on nematode populations and soil health is needed to optimize small grain winter cover crops in integrated pest management programs.

In Southeastern U.S. cotton production, there is a need for more sustainable management strategies for *M. incognita* and *R. reniformis* that effectively reduce nematode populations while supporting optimal plant growth and yield. Crop rotations with cover crops are effective in naturally disrupting disease cycles for plant-parasitic nematodes that may reduce the intensive use of herbicides and pesticides (Phatak and Diaz-Perez, 2012). Studies quantifying *M. incognita* reproduction levels on small grain and legume cover crops in cotton production fields determined that small grains are more effective in limiting *M. incognita* reproduction (Timper et al., 2006; Wang et al., 2004). Similarly, research shows that small grain cover crops (rye, wheat, and black oat) support lower population density of *R. reniformis* compared to some legume cover crops (Jones et al., 2006). Strategically incorporating small grain cover crops into an integrated nematode management plan is an environmentally conscious strategy for maintaining *M. incognita* and *R. reniformis* level below their damage thresholds (Roberts, 1993). Furthermore, small grains can be used for livestock grazing or harvested for grain that may allow growers to maximize their land use and create new sources of income (Lee et al., 2017; Tyson and Hammond, 2017).

*Meloidogyne incognita* ([Kofoid and White] Chitwood) is a major pest of cotton and is found in all cotton producing states in the U.S. (Thomas and Kirkpatrick, 2001). It feeds as a sedentary endoparasite, blocking the passage of water and nutrients within the vascular tissue of the host plant (Lambert and Bekal, 2002). Root infection by *M. incognita* results in the formation of large galls on the plant’s root system, which is the most diagnostic symptom of *M. incognita* damage (Jones et al., 2013). Reduced water and nutrient uptake contribute to the development of above-ground symptoms, such as wilting, stunting, yellowing and yield reduction (Lawrence and Lawrence, 2020). *Meloidogyne incognita* requires at least 18 °C to remain active in the soil but can develop and reproduce at temperatures as low as 10 °C if root infection has already taken place (Roberts et al., 1981). It has an extensive host range of over 2,000 plant species among important agronomic and horticultural crops and various weed species, which makes management efforts challenging (Starr et al., 2007). Effective management of *M. incognita* is largely dependent on synthetic nematicides, which constitutes a threat for environmental and human safety. The integration of cover crops may be an alternative for managing plant-parasitic nematodes that may alleviate the exhaustive use of nematicides.

*Rotylenchulus reniformis* (Linford and Oliveira) is increasingly problematic throughout the U.S. cotton belt. It is well-adapted to tropical and subtropical regions and soils with high silt and clay concentrations (Kinloch and Sprenkel, 1994). *Rotylenchulus reniformis* feeds on plant roots as a semi-endoparasite that causes injury to cotton plants producing symptoms, such as irregular and stunted plant growth, limited root development, and reduced boll size and yield (Lawrence and Lawrence, 2020). In most temperate climates, *R. reniformis* requires at least 25 °C to reproduce; however, in more tropical regions, it can reproduce at 15 °C with adequate soil moisture (Heald and Inserra, 1988). Under optimal conditions, *R. reniformis* can complete its life cycle within 25 to 30 days (Birchfield, 1962). Its rapid reproduction rate can cause *R. reniformis* to be a severe a problem for growers if not properly managed (Greer et al., 2009). Growers rely heavily on nematicides to reduce *R. reniformis* population levels in cotton production fields (Starr et al., 2007). There is a need for alternative management strategies that are less of a risk for sustainable agriculture but allow growers to maintain their current production levels.

Crop rotation with cover crops is a critical management tool that can help reduce nematode population levels by outcompeting weeds that may serve as alternate hosts for plant-parasitic nematodes and creating a suitable environment for soil microorganisms that can be antagonistic to these soil-borne pathogens (Phatak and Diaz-Perez, 2012). Furthermore, there are numerous benefits provided by cover crops beyond nematode management that can improve the physical characteristics of the soil (Price et al., 2008; Town et al., 2022). Small grain cover crops are favored in the Southeast compared to other cover crop options due to their greater cold tolerance and abundant above-ground biomass that protects the soil structure and improves soil quality (SARE, 2012).

Cover crops are primarily defined by their use for soil health benefits. The USDA specifically defines cover crops as being used primarily for erosion control, soil health improvement, and water quality enhancement (USDA-NRCS, 2019). This distinction is crucial, as cover crops are not considered ‘crops’ for insurance purposes (Wallander et al., 2021). The adoption of cover crops has been substantially driven by financial incentive programs offered at both the federal and state levels. In 2018, approximately one-third of the acreage planted with cover crops received financial assistance payments from various programs (Wallender et al., 2021). Between 2011 and 2015, the total acreage enrolled in the USDA’s Conservation Stewardship Program (CSP) for cover crop practices increased from approximately 350,000 acres to more than 2 million acres (Wallander et al., 2021). Additionally, state-level incentive programs supported over 1 million acres of cover crops in 2018 across at least 22 states (Wallander et al., 2021).

While cover crops are primarily grown for soil health benefits, there is potential for dual-purpose use with small grain cover crops. According to USDA-NRCS (2019) guidelines, cover crops may be grazed or harvested as hay or silage, unless prohibited by specific crop insurance policy provisions. However, they cannot be harvest for grain or seed under these programs (USDA-NRCS, 2019). For growers not participating in financial assistance programs, there may be opportunities to optimize small grain cover crops for grain. This flexibility could provide additional economic benefits to growers while still maintaining the soil health advantages of cover cropping. By carefully selecting varieties, adjusting planting dates, and managing fertility, growers can potentially harvest a marketable grain crop while preserving the soil-improving qualities of cover crops. This strategy can diversify income streams and make more efficient use of land throughout the year (Schipanski et al., 2014).

The purpose of this experiment was to assess the capability of small grain cover crops to reduce *M. incognita* and *R. reniformis* population density in environments in the Southeast that traditionally experience warm winter temperatures, which may support nematode reproduction on these winter cover crops. The objectives of this research were 1) to determine the effectiveness of small grain cover crops as an additional sustainable nematode management strategy by measuring reproduction of *M. incognita* and *R. reniformis* on fall-planted small grains in cotton production fields; and to 2) analyze the forage quality and quantify the grain yield of the small grain cultivars to assess their potential as dual-purpose crops.

## Materials and Methods

### Greenhouse experiments

*Nematode inoculum*: *Meloidogyne incognita* race 3 used for inoculum in the greenhouse experiments was prepared at the Plant Science Research Center (PSRC) in Auburn, AL, from stock cultures maintained on DEKALB DKC68-26 corn in 500 cm^3^ polystyrene pots. *Meloidogyne incognita* eggs were extracted from the corn roots by agitating the root systems in a 0.625% NaOCl solution on a Barnstead Lab Line Max Q 5000 E class shaker for 4 min at 120 rpm (Conquer Scientific: San Diego, CA) (Hussey and Barker, 1973). Roots were then washed under tap water and eggs were collected on a 25-μm-pore sieve. The contents collected from the 25-μm-pore sieve were processed by a modified sucrose centrifugation-flotation at 240 g-forces for 1 minute (Jenkins, 1964). An inverted TS100 Nikon^®^ microscope was used at 40x magnification to confirm the presence of *M. incognita* and enumerate eggs to a standardized 2,000 eggs/ml where 1 ml was pipetted into each 150 cm^3^ cone-tainer 7 DAE of small grain and corn plants in the greenhouse.

Advanced breeding lines from OreGro Seeds, INC. (Albany, OR) and commercial varieties across four small grain crop groups were tested with *M. incognita* to determine the susceptibility of the small grain cover crops in the greenhouse. The four small grain groups tested included eight triticale (x *Triticosecale* Wittmack), five barley (*Hordeum vulgare*), four wheat (*Triticum aestivum*), and three oat (*Avena sativa*) cultivars in comparison to DEKALB DKC68-26 corn (*Zea mays*) for *M. incognita* host susceptibility. The A Kalmia loamy sand soil (80% sand, 10% silt, 10% clay) acquired from the Plant Breeding Unit (PBU) of the E.V. Smith Research Center (EVS) near Tallassee, AL, was used for all experiments. The soil was steam pasteurized at 80 °C for 90 minutes and cooled for 24 hours; the process was repeated once more to prevent the regeneration of potential plant pathogens. The pasteurized soil was mixed with sand to a combined ratio of 60:40, soil to sand. Prior to use, fertilizer and lime were added to the soil according to recommendations specified by the Auburn University soil, forage, and water testing laboratory. All tests were performed in 150 cm^3^ plastic cone-tainers (Stuewe & Sons, Inc., Tangent, OR). Four seeds of each variety of small grains and two seeds of DKC68-26 corn were planted 1 cm deep in each cone-tainer. Small grain plants were thinned to two plants per cone after germination. Tests were planted in early and again in late summer with greenhouse temperatures ranging from 24 °C to 35 °C, and supplemental lighting was supplied via 1000-watt halide bulbs producing 110,000 lumens at a rate of 14 hours per day. Plants were watered as needed to maintain soil moisture between 40% and 60%. All plants were inoculated with *M. incognita* eggs 7 days after emergence (DAE).

*Experimental design*: The small grain cultivars *M. incognita* host status tests were arranged in a randomized complete block design (RCBD) with five replications and the experiment was repeated. The early and late summer greenhouse experiments were terminated 42 days after planting. Timing of germination was not recorded. Plant height and root fresh weight were collected for each experiment. When *M. incognita* was present, total nematode eggs per cone-tainer and eggs per gram of root were also recorded. *Meloidogyne incognita* eggs were extracted from small grain and corn roots and enumerated as previously described. Reproduction factor was determined as Rf = Pf (final population)/Pi (initial population) as specified by Oostenbrink (1966). The Rf values were grouped into four categories as follows: Rf=0–0.09, nonhost; Rf=0.1–0.9, poor host; Rf=1-2, moderate host; Rf>2, suitable host (Oostenbrink, 1966).

### Field experiments

**Figure 1:**
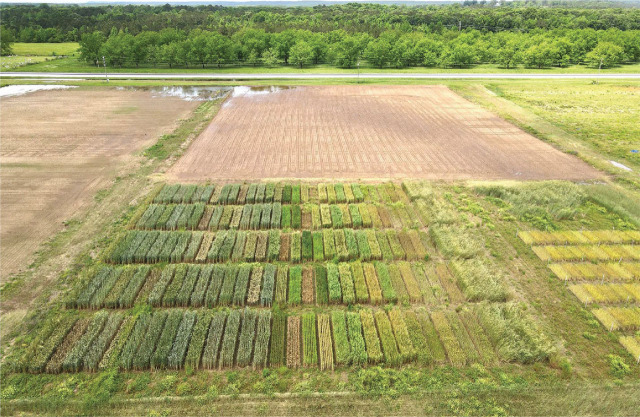
Thirty-one winter small grain cultivars arranged in a randomized complete block design, photographed on 4 May 2021 in the *Meloidogyne incognita* field at the Plant Breeding Unit near Tallassee, Alabama.

**Figure 2:**
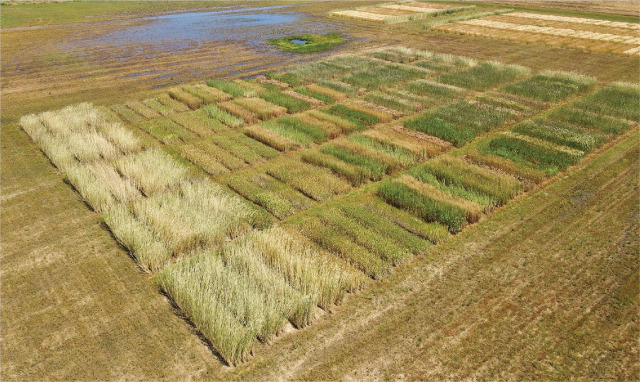
Thirty-one winter small grain cultivars arranged in a randomized complete block design, photographed on 6 May 2021 in the *Rotylenchulus reniformis* field at the E.V. Smith Research Center near Shorter, Alabama.

Field experiments examining small grain winter cover crops were conducted from 2019 to 2021 under *M. incognita* stress at the PBU in Tallassee, AL (Fig. 1) and under *R. reniformis* stress at the EVS in Shorter, AL (Fig. 2). These experiments analyzed a larger selection of small grain cover crops than what was tested in the greenhouse, including 14 triticale, 5 barley, 5 oat, 4 wheat, and 3 rye (*Secale cereale* L.) cultivars. The small grain cultivars were a combination of advanced breeding lines from OreGro Seeds, INC. (Albany, OR) and additional commercial cultivars. Both field experiments were maintained by research station personnel throughout the winter growing season. Small grain winter cover crop experiments were established in fields previously grown in cotton. PBU is naturally infested with *M. incognita* and has a Kalmia loamy sand soil (80% sand, 10% silt, 10% clay) classification. The *M. incognita* population of J2 was 52/100 cm^3^ of soil and 682 J2/100 cm^3^ of soil in 2019 and 2020, respectively. At EVS, fields are naturally infested with *R. reniformis,* and the soil is a Compass loamy sand (76% sand, 13.6% silt, 10.4% clay). The *R. reniformis* total vermiform life stage numbers was 511/100 cm^3^ of soil in 2019 and 981/100 cm^3^ of soil in 2020. In all field experiments, the small grain cultivars were organized within their respective crop group and were arranged in a RCBD with 5 replications. Prior to planting, the seedbed was prepared with a KMC field cultivator and a Lely roterra tiller. The small grain cover crops were drilled at 100 grams of seed per plot with a Hege field plot grain drill (Hege Equipment Inc., Colwich, KS) at 19.1 cm row spacing. Individual plots were 6.1 m in length and 1.2 m in width with 3 m alleys in between replications. The 31 small grain cultivars were sown in fields with *M. incognita* at the PBU on November 20, 2019, and November 13, 2020. The same small grain cultivars were established at EVS in fields with *R. reniformis* on October 3, 2019, and November 16, 2020. Fertility management in both years at both locations included an application of 17-17-17 at 14 kg/ha on the same day as planting prior to drill-seeding. A broadcast application of 33-0-0 was applied in mid-February in both years at the PBU. At EVS, 33-0-0 was broadcasted in late January during the 2019–2020 growing season and in mid-February during the 2020–2021 growing season. Grain was harvested using an ALMACO R1 rotary single plot combine (Nevada, Iowa). In the 2019–2020 growing season, grain was harvested on June 12, 2020, at the PBU, and on May 21, 2020, at EVS. In the 2020–2021 growing season, grain was harvested on June 17, 2021, at both locations.

*Field experimental design*: To quantify the pre-planting population density of *M. incognita* and R. *reniformis* to a potential subsequent cotton crop, four random plant samples representative of each plot were collected. In the first trial year, plant and root samples were collected from both PBU and EVS on May 11, 2020. In the second trial year, plant and root samples were collected from PBU on May 6, 2021, and from EVS on May 18, 2021. The four random plant samples were manually dug with a shovel from each plot. From these plant and root samples, measurements including, plant height, root fresh weight, and nematode eggs per gram of root were recorded. Plant and root samples were processed for nematodes as described in the greenhouse experiments. To compare the biomass yield between the small grain winter cover crops, a 30-cm square was randomly placed in each plot and the above-ground biomass was collected, leaving an 8-cm stubble, and then weighed. Biomass samples were not dried before weighing. In the 2019–2020 growing season at both locations, above-ground biomass cuttings were taken near harvest on May 11, 2020. In the 2020–2021 growing season, above-ground biomass cuttings were taken on May 6, 2021, at the PBU, and on May 18, 2021, at EVS.

*Forage quality field experiments:* Field experiments were established at an on-farm location near Germanton, NC, in a region of the Southeast that is more adapted for cool-season annual grain production. The same 31 small grain cultivars tested in the nematode field experiments were planted in North Carolina on October 19, 2019, and November 25, 2020, in a field that was previously sown in tall fescue. These small grains were measured for plant height, biomass yield, and forage quality. The experiments were managed by the grower at the on-farm site. Prior to planting in the 2019–2020 growing season, 3.36 kg/ha of glyphosate was applied to the field trial site to eliminate the established tall fescue crop, and the seedbed was then prepared with a Krause 2800 disk chisel followed by a Taylor-Way 590 Tandem Disc Harrow and Brillion Cultipacker. In both years, a pre-plant application of 17-17-17 and then a mid-season topdressing of 46-0-0 in late February were applied. The soil classification at this on-farm site is a Codorus loam (25% clay, 30% sand, 45% silt). In both experiments, the small grain cultivars were organized within their respective crop group and were arranged in a RCBD with 5 replications. The field experiments were seeded with a Clean Seeder AP 2-line push planter (Sutton Ag Enterprises, Salinas, CA) at 15.2 cm row spacing. Individual plots were 6.1 m in length and 1.2 m in width with 1.5 m alleys in between replications. The grazing potential of these small grains was measured by biomass yield and forage quality analyses. Forage samples were collected by placing, randomly, a 30-cm square in each plot and cutting the biomass within the square leaving an 8-cm stubble. For each small grain cultivar, the biomass cuttings from all replications were combined for a representative sample and weighed. Biomass samples were not dried before weighing. One pound of the composite sample was packaged in a plastic bag and frozen before being shipped to the Dairy One Forage Lab (Ithaca, NY) for forage quality analysis. The forage quality of these small grain cover crops during the vegetative state was analyzed on April 15 and May 22 in 2020 and on April 15 and May 14 in 2021, and compared based on crude protein, acid detergent fiber (ADF), neutral detergent fiber (NDF), total digestible nutrients (TDN), and relative feed value (RFV). These criteria were used to evaluate the overall nutritive value of the small grains.

*Statistical analysis:* Data collected from the greenhouse and field experiments were analyzed in SAS 9.4 (SAS Institute, Cary, NC) using the PROC GLIMMIX procedure. Dependent variables included plant height, root fresh weight, *M. incognita* and *R. reniformis* eggs per gram of root, biomass yield (kg/ha), and grain yield (kg/ha). Random effects included replication and test repeats. The small grain winter forage crop tests found no significant interactions between repeated trials in the greenhouse and two years of the field tests, and thus, data from the repeated trials for each experiment were combined for analysis. LS means were compared between individual cultivars using ANOVA and Tukey-Kramer multiple pairwise comparison at a significance level of *P* ≤ 0.05.

## Results

*Greenhouse experiments*: The greenhouse experiments demonstrated that the average *M. incognita* eggs per gram of root were greater in cone-tainers containing the small grains (triticale, wheat, oat, and barley) compared to the DK68-26 corn variety (Table 1). The average Rf values for cultivars of oat and triticale were 1.13 and 1.86, respectively, which were considered to be moderate hosts of *M. incognita* in the greenhouse setting. Barley and wheat cultivars averaged Rf values of 2.44 and 2.99, respectively, measuring to be suitable hosts in the greenhouse (Table 1). Three small grain cultivars, ‘Forerunner’ and ‘OG170039’ triticale and ‘ORO 4372’ oat, had Rf less than one, indicating that these specific cultivars were poor hosts for *M. incognita* (Table 1). All other cultivars tested among the small grain crops were determined to be either moderate or suitable hosts (Table 1).

**Table 1:** Host susceptibility of *Meloidogyne incognita* on commercial cultivars and developmental lines of winter small grains tested under greenhouse conditions at the Plant Science Research Center in Auburn, AL measured by average number of eggs per gram of root and reproductive factors.

	**Plant Science Research Center**		

**Cultivar**	***M. incognita***		
	**Eggs/g Root^v^**	**Rf Value^w^**	**Host Suitability**
**Triticale (x *Triticosecale* Wittmack)**			
Doublet^y^	2015 c^z^	2.02^x^	Suitable Host
Forerunner^y^	183 g	0.38	Poor Host
EST 2640	2473 b	2.89	Suitable Host
EST 2767	628 e	1.35	Moderate Host
EST 2824	2606 c	1.5	Moderate Host
ORO 4370	2420 d	3.01	Suitable Host
ORO 4371	4366 a	2.95	Suitable Host
OG170039	393 f	0.75	Poor Host
***Crop Average***	***1886***	***1.86***	
***Wheat (Triticum aestivum)***			
KGAL^y^	3579 a	3.68	Suitable Host
Summit 515^y^	3761 a	2.86	Suitable Host
Willow Creek^y^	1038 b	1.48	Moderate Host
ORO 4373	8773 a	3.93	Suitable Host
***Crop Average***	***4288***	***2.99***	
**Oat (*Avena sativa*)**			
Intimidator^y^	682 b	1.08	Moderate Host
Shooter^y^	1021 a	1.42	Moderate Host
ORO 4372	1666 c	0.89	Poor Host
***Crop Average***	***1123***	***1.13***	
**Barley (*Hordeum vulgare*)**			
Alba^y^	476 d	1.61	Moderate Host
Verdant^y^	403 e	1.11	Moderate Host
OG140760	5513 a	3.53	Suitable Host
OG140789	2155 b	2.84	Suitable Host
OG140797	1760 c	3.10	Suitable Host
***Crop Average***	***2061***	***2.44***	
**Corn (*Zea mays*)**			
DKC68-26	**187**	**1.76**	Moderate Host

z LS-means followed by the same letter are not significantly different at *P* ≤ 0.05 as determined by the Tukey-Kramer method. Data are from two tests combined for a total of 10 replications per small grain.

y Indicates commercial small grain cultivar.

x Rf= final population/initial population of *M. incognita*.

w Rf values are grouped as follows: Rf=0–0.09, nonhost; Rf=0.1–0.9, poor host; Rf=1-2, moderate host; Rf>2, suitable host.

v Small grain cultivars were statistically analyzed within their respective crops.

*Meloidogyne incognita field seasons 2019–2020 and 2020–2021*: In samples taken near harvest in the *M. incognita* field, rye cultivars had the greatest plant height of the small grain crops tested, followed by oat and triticale cultivars, respectively (Table 2). Barley and wheat cultivars, on average, were shorter than rye, oat, and triticale cultivars (Table 2). ‘Abruzzi’ rye was the tallest of the rye cultivars followed by ‘Goku’ and ‘Elbon’, respectively (Table 2). ‘Shooter’ oat, had the greatest plant height of the oat cultivars and was significantly greater than ‘Intimidator’, ‘OG6285’, and ‘Buck Forage’, respectively, of which had statistically similar plant heights to each other (Table 2). ‘TAMO 411’ was the shortest plant of the oat cultivars (Table 2). For triticale cultivars, the plant height measurements near harvest were statistically similar except for ‘158 EP’, which was significantly shorter (Table 2). ‘OG140760’ was the tallest barley cultivar but was statistically comparable to ‘OG140797’ and ‘Verdant’, respectively (Table 2). ‘Alba’ and ‘OG140789’ barley cultivars had statistically similar plant heights but were significantly the shortest of the barley cultivars (Table 2). The tallest wheat cultivars were ‘KGAL’ and ‘Willow Creek’, respectively, followed by ‘OG9484’ and ‘Summit 515’, respectively, which were significantly shorter than ‘KGAL’ and ‘Willow Creek’ (Table 2).

**Table 2:** Plant height, dry matter, grain yield, and total seasonal *Meloidogyne incognita* population numbers on small grain cover crops planted on 20 November 2019 and 13 November 2020 at the Plant Breeding Unit near Tallassee, AL.

**Plant Breeding Unit 2019–2021**				

**Cultivar^x^**	**Plant Height (cm)**	**Fresh Biomass Weight (kg/ha)**	**Grain Yield (kg/ha)**	**Total Seasonal *M. incognita* (eggs/g root)**
**Triticale (x *Triticosecale* Wittmack)**				
Doublet^y^	110 dc^z^	21774	4516 a	5
Forerunner^y^	125 abc	16436	1547 b	3
Round Table^y^	113 bcd	10572	1586 b	1
158 EP^y^	75 e	4947	3028 ab	2
OG8782	96 de	6500	2250 b	3
OG8783	135 ab	17008	2781 b	4
OG170004	132 abc	20387	1827 b	2
OG170012	146 a	19879	1942 b	2
OG170023	129 abc	24542	2146 b	3
OG170035	138 a	22057	2070 b	1
OG170036	132 abc	22037	2110 b	5
OG170039	128 abc	17457	2235 b	2
OG170040	134 ab	22564	1928 b	2
OG170043	131 abc	16744	1966 b	1
***Crop Average***	***123***	***17350***	***2281***	***3***
**Wheat (*Triticum aestivum*)**				
KGAL^y^	92 a	23670	2117 ab	2
Summit 515^y^	65 c	3257	829 b	3
Willow Creek^y^	92 a	14612	787 b	3
OG9484	79 b	12457	3077 a	4
***Crop Average***	***82***	***13499***	***1702***	***3***
**Oat (*Avena sativa*)**				
Buck Forage^y^	129 b	15917	6192	6
Intimidator^y^	135 b	30606	1293	4
Shooter^y^	153 a	47559	2667	2
TAMO 411^y^	106 c	30147	4707	3
OG6285	130 b	25244	1992	5
***Crop Average***	***131***	***29895***	***3370***	***4***
**Barley (*Hordeum vulgare*)**				
Alba^y^	69 b	14629	2816	6
Verdant^y^	82 a	17129	2007	10
OG140760	89 a	17735	2456	18
OG140789	64 b	20879	1881	10
OG140797	83 a	20733	2231	6
***Crop Average***	***77***	***18221***	***2278***	***10***
**Rye (*Secale cereale*)**				
Abruzzi^y^	174	35830	2464	1
Elbon^y^	154	26665	1797	3
Goku^y^	161	23154	2303	3
***Crop Average***	***163***	***28550***	***2188***	***2***

z LS-means that share the same letter are not significantly different at P ≤ 0.05, according to the Tukey-Kramer test. If no letters follow a data point, it indicates that no significant differences were detected.

y Indicates commercial small grain cultivar.

x Small grain cultivars were statistically analyzed within their respective crop.

For all small grain cultivars, biomass yield near harvest ranged from 3,257 to 47,559 kg/ha (Table 2). Biomass yield was numerically greater in the oat cultivars followed by rye, barley, triticale, and wheat cultivars, respectively (Table 2).

Overall, oat cultivars had significantly greater yields compared to all other small grain cultivars, followed by triticale, barley, rye, and wheat, respectively (Table 2). Grain yield for all small grain cultivars ranged from 787 to 6,192 kg/ha (Table 2). All oat cultivars yielded statistically similar kg/ha with ‘Buck Forage’ supporting the greatest yield (Table 2). Within triticale, ‘Doublet’ yielded the greatest kg/ha followed by ‘158 EP’, ‘OG8783’, and ‘OG8782’, respectively (Table 2). The remaining triticale cultivars had statistically similar yields to each other and were comparable to ‘158 EP’, ‘OG8783’, and ‘OG170039’, but yielded significantly less kg/ha than ‘Doublet’ (Table 2). The barley cultivars had statistically similar yields with ‘Alba’ supporting the greatest yield followed by ‘OG140760’, ‘OG140797’, ‘Verdant’, and ‘OG140789’ (Table 2). ‘Abruzzi’ had the greatest yield of the rye cultivars followed by ‘Goku’ and ‘Elbon’, respectively, and all rye cultivars had statistically similar yields (Table 2). All rye cultivars, ‘Abruzzi’, ‘Goku’, and ‘Elbon’, respectively, yielded statistically similar kg/ha (Table 2). Within wheat, ‘OG9484’ yielded the greatest kg/ha followed by ‘KGAL’ (Table 2). The remaining wheat cultivars, ‘Summit 515’ and ‘Willow Creek’, respectively, had statistically similar yields to each other and ‘KGAL’, but were significantly shorter than ‘OG9484’ (Table 2).

The total seasonal *Meloidogyne incognita* numbers per gram of root were statistically similar between the cultivars in each small grain group (Table 2). Barley cultivars supported the greatest total seasonal *M. incognita* per gram of root followed by oat, wheat, triticale, and rye, respectively (Table 2).

*Rotylenchulus reniformis field seasons 2019–2020 and 2020–2021I*: In the *Rotylenchulus reniformis* field experiment, rye cultivars had the greatest plant height followed by oat, triticale, barley, and wheat, respectively (Table 3). ‘Abruzzi’ was the tallest rye cultivar followed by ‘Goku’ and ‘Elbon’, respectively, and all rye cultivars had statistically similar plant height (Table 3). Within the oat cultivars, ‘Shooter’ was the tallest on average followed by ‘Buck Forage’ and ‘OG6285’, respectfully, which were statistically similar to each other, but significantly taller than ‘Intimidator’ and ‘TAMO 11’, respectively (Table 3). All triticale cultivars had statistically comparable plant height, except for ‘OG8782’ and ‘158 EP’, respectively, which were significantly shorter (Table 3). For barley, ‘OG140760’ had the greatest plant height of the barley cultivars followed by ‘Alba’, ‘OG140797’, and ‘Verdant’, respectively (Table 3). ‘OG140789’ was significantly shorter on average than all other barley cultivars (Table 3). Within the wheat cultivars, ‘KGAL’ and ‘OG9484’ had the greatest plant height followed by ‘Willow Creek’ (Table 3). ‘Summit 515’ had statistically similar plant height to ‘Willow Creek’ but was significantly shorter than ‘KGAL’ and ‘OG6285’ (Table 3).

**Table 3:** Plant height, dry matter, grain yield, and total seasonal *Rotylenchulus reniformis* population numbers on small grain cover crops planted on 3 October 2019 and 16 November 2020 at E.V. Smith Research Center near Shorter, AL.

**E.V. Smith Research Center 2019–2021**				

**Cultivar^x^**	**Plant Height (cm)**	**Fresh Biomass Weight (kg/ha)**	**Grain Yield (kg/ha)**	**Total Seasonal *R. reniformis* (eggs/g root)**
**Triticale (x *Triticosecale* Wittmack)**				
Doublet^y^	101 b^z^	18790	1979	4
Forerunner^y^	116 ab	14307	1010	12
Round Table^y^	100 b	13668	748	5
158 EP^y^	69 c	8243	1381	5
OG8782	77 c	7618	1627	4
OG8783	117 ab	17677	1633	4
OG170004	119 a	14234	1069	2
OG170012	127 a	23228	1324	6
OG170023	120 a	14449	1037	7
OG170035	125 a	18912	1364	6
OG170036	119 a	18340	1239	3
OG170039	120 a	16876	1331	7
OG170040	119 a	17862	1061	6
OG170043	117 ab	17310	1346	5
***Crop Average***	***110***	***15822***	***1296***	***5***
**Wheat (*Triticum aestivum*)**				
KGAL^y^	85 a	19826	1154	3
Summit 515^y^	51 b	4659	755	5
Willow Creek^y^	65 ab	12621	1304	2
OG9484	84 a	11836	2126	3
***Crop Average***	***71***	***12235***	***1335***	***3***
**Oat (*Avena sativa*)**				
Buck Forage^y^	135 a	20768	1248 b	6
Intimidator^y^	114 b	21105	861 b	7
Shooter^y^	142 a	22853	984 b	8
TAMO 411^y^	110 b	19386	3151 a	3
OG6285	132 a	13839	1359 b	5
***Crop Average***	***127***	***19590***	***1521***	***6***
**Barley (*Hordeum vulgare*)**				
Alba^y^	74 b	9669	2171 a	10
Verdant^y^	71 b	9518	1256 b	6
OG140760	85 a	9801	1887 ab	4
OG140789	57 c	11866	1412 ab	7
OG140797	72 b	11612	1657 ab	5
***Crop Average***	***72***	***10493***	***1677***	***6***
**Rye (*Secale cereale*)**				
Abruzzi^y^	149	17467	2281a	7
Elbon^y^	140	11905	1405b	7
Goku^y^	145	10099	1178b	4
***Crop Average***	***144***	***13157***	***1621***	***6***

z LS-means that share the same letter are not significantly different at *P* ≤ 0.05, according to the Tukey-Kramer test. If no letters follow a data point, it indicates that no significant differences were detected.

y Indicates commercial small grain cultivar.

x Small grain cultivars were statistically analyzed within their respective crop.

For all small grain cultivars, biomass yield near harvest ranged from 4,659 to 23,228 kg/ha (Table 3). Biomass yield was numerically greater in the oat cultivars followed by triticale, rye, wheat, and barley, respectively (Table 3).

Barley cultivars supported the greatest grain yield followed by rye, oat, wheat, and triticale cultivars, respectively (Table 3). ‘Alba’ barley yielded the greatest kg/ha followed by ‘OG140760’, ‘OG140797’, and ‘OG140789’, respectively, which were statistically similar to ‘Alba’ (Table 3). ‘Verdant’ yielded the lowest kg/ha and was significantly shorter than ‘Alba’, but statistically similar to all other barley cultivars (Table 3). ‘Abruzzi’ had the greatest grain yield for rye cultivars followed by ‘Elbon’ and ‘Goku’, respectively, which had statistically similar yields (Table 3). Within the oat cultivars, ‘TAMO 411’ had a significantly greater yield of all oat cultivars followed by ‘OG6285’, ‘Buck Forage’, ‘Shooter’, and ‘Intimidator’, respectively. There was no significant difference in grain yield for cultivars of wheat and triticale (Table 3).

The total seasonal *R. reniformis* numbers per gram of root were statistically similar between the cultivars in each small grain group (Table 3). Oat, barley, and rye cultivars supported equal and the greatest total seasonal *R. reniformis* per gram of root, followed by triticale and wheat, respectively (Table 3).

*Forage quality 2019–2020 and 2020–2021*: In the forage quality experiments in North Carolina, rye cultivars were the tallest small grain followed by oat, triticale, barley, and wheat, respectively (Table 4). There were no significant differences in plant height within the cultivars of rye and of oat (Table 4). All triticale cultivars had statistically similar plant height except for ‘OG8782’ and ‘158 EP’, respectively, which were significantly shorter (Table 4). Within the barley cultivars, “OG140760’ had the greatest plant height and was statistically similar to ‘OG140797’ and ‘Verdant’ but was significantly greater than ‘Alba’ and ‘OG140789’ (Table 4). ‘OG140789’ was significantly the shortest of all barley cultivars (Table 4). For wheat, ‘Willow Creek’ supported the greatest plant height followed by ‘KGAL’, which had statistically similar plant height (Table 4). ‘OG9484’ and ‘Summit 515’ had statistically similar plant height to ‘KGAL’ but were significantly shorter than ‘Willow Creek’ (Table 4).

**Table 4:** Plant height, fresh biomass yield and forage quality composite samples analysis of small grain cover crops in the on-farm forage trial in Germanton, NC, in the cool seasons of 2019–2020 and 2020–2021.

**Cultivar**	**Plant Height (cm)**	**Fresh Biomass Weight (kg/ha)**	**% CP ^x^^y^**	**% ADF ^w^^y^**	**% NDF v^y^**	**%TDN ^u^^y^**	**RFV^t^^y^**
**Triticale (*x Triticosecale* Wittmack)**							
Doublet^z^	118 bc^y^	4060	20.85	27.75	48.15	68.50	131
Forerunner^z^	125 bc	4283	24.30	26.30	45.90	67.50	140
Round Table^z^	116 bc	3477	20.35	28.40	50.95	67.00	123
158 EP^z^	82 d	3893	20.40	28.55	47.95	67.00	135
OG8782	97 d	5730	18.15	31.40	53.10	65.00	116
OG8783	143 ab	6350	21.70	28.30	48.40	68.50	132
OG170004	142 ab	5499	23.75	26.00	44.55	67.50	143
OG170012	157 a	5386	22.50	26.30	45.30	68.00	141
OG170023	141 ab	3923	23.05	25.65	41.45	69.50	155
OG170035	132 ab	4422	25.45	25.70	45.10	68.00	142
OG170036	135 ab	4379	22.35	26.60	44.55	69.50	143
OG170039	140 ab	5416	24.40	25.35	42.35	68.00	152
OG170040	136 ab	4828	25.85	27.35	42.05	66.00	150
OG170043	131 ab	4474	22.80	24.80	42.65	68.50	152
***Crop Average***	***128***	***4723***	***22.56***	***27.03***	***45.89***	***67.75***	***139***
**Wheat (*Triticum aestivum*)**							
KGAL^z^	100 ab	3578	23.75	24.55	41.45	68.50	158
Summit 515^z^	75 b	3983	19.50	30.65	52.90	64.00	119
Willow Creek^z^	119 a	3077	28.10	25.90	43.45	66.00	147
OG9484	86 b	3048	22.70	26.05	43.30	66.00	148
***Crop Average***	***95***	***3421***	***23.51***	***26.79***	***45.28***	***66.13***	***143***
**Oat (*Avena sativa*)**							
Buck Forage^z^	134	5535	22.65	24.85	42.65	70.00	152
Intimidator^z^	134	4915	18.20	25.65	43.80	71.00	148
Shooter^z^	139	6066	18.85	25.85	45.20	70.50	142
TAMO 411^z^	111	7128	19.85	25.10	43.90	70.50	148
OG6285	131	5048	19.70	23.20	39.30	70.50	168
***Crop Average***	***130***	***5738***	***19.85***	***24.93***	***42.97***	***70.50***	***151***
**Barley (*Hordeum vulgare*)**							
Alba^z^	102 b	5474	18.15	25.50	44.10	69.50	146
Verdant^z^	108 ab	5311	18.05	25.70	44.65	70.00	144
OG140760	113 a	4895	17.80	23.90	42.70	71.00	153
OG140789	91 c	4496	18.85	24.10	42.70	70.00	153
OG140797	109 ab	4378	19.45	25.80	43.85	70.00	146
***Crop Average***	***104***	***4911***	***18.46***	***25.00***	***43.60***	***69.70***	***148***
**Rye (*Secale cereale*)**							
Abruzzi^z^	166	9117	18.20	2720	49.30	68.50	128
Elbon^z^	166	8656	19.20	25.75	46.00	67.50	141
Goku^z^	169	8042	16.70	28.25	48.50	69.50	130
***Crop Average***	***167***	***8605***	***18.03***	***27.07***	***47.93***	***68.50***	***133***

z Indicates commercial small grain cultivar.

y LS-means that share the same letter are not significantly different at P ≤ 0.05, based on the Tukey-Kramer test. The absence of letters following a data point indicates that no significant differences were detected. Forage quality analysis was conducted on a composite sample across replications and was not subjected to statistical analysis.

x % CP refers to percent of crude protein and is measured by nitrogen content. % CP of cool-season grasses varies between 8–23 %.

w % ADF refers to percent of acid detergent fiber. Lower % ADF indicates better digestibility. Depending on plant maturity, most forages have % ADF between 24–51 %.

v % NDF refers to percent of neutral detergent fiber. Lower % NDF indicates higher intake. Depending on plant maturity, most forages have % NDF between 29–66 %.

u % TDN refers to percent of total digestible nutrients. Cool-season grasses should have TDN values between 55–68%.

t RFV refers to relative feed value and is an index that ranks quality from prime (highest) through grade 5 (lowest); Prime:>151, Grade 1:125–151, Grade 2:103–124, Grade 3:87–102; Grade 4:75–86; Grade 5:<75.

For all small grain cultivars, biomass yield in the vegetative phase ranged from 3,048 to 7,128 kg/ha (Table 3). Biomass yield in the vegetative phase was numerically greater in the oat cultivars followed by triticale, rye, wheat, and barley, respectively (Table 3). Overall, crude protein (CP) concentrations of the small grain crops ranged from 17.8% to 28.1 % (Table 4). The highest CP concentrations were supported by wheat cultivars, followed by triticale, oat, barley, and rye, respectively (Table 4). Percent ADF for all small grain crops ranged from 23.2% to 31.4 % (Table 4). Rye and triticale cultivars had the highest and almost equal percent ADF followed by wheat, barley, and oat, respectively (Table 4). Percent NDF for all small grain crops ranged from 39.3% to 53.1 % with the highest NDF concentrations supported by rye cultivars followed by triticale, wheat, barley, and oat, respectively (Table 4). Percent TDN for all small grain crops ranged from 64% to 71 % (Table 4). Oat cultivars had the greatest % TDN followed by barley, rye, triticale, and wheat, respectively (Table 4). The RFV index for all small grain crops ranged from 116 to 168 (Table 4). The highest RFV indices were supported by oat cultivars followed by barley, wheat, triticale, and rye, respectively (Table 4).

## Discussion

*Greenhouse experiment*: All small grain cultivars evaluated in the greenhouse supported higher average *M. incognita* eggs per gram of root compared to the standard DK68-26 corn variety that was included in the tests as a standard comparison. The Rf values revealed varying levels of host suitability for *M. incognita* among the small grain cultivars. Overall, barley and wheat demonstrated high suitability as host plants, while triticale and oat exhibited moderate host potential. Notably, two triticale cultivars, ‘Forerunner’ and ‘OG170039’ and one oat cultivar, ‘ORO 4372’ stood out as poor hosts, exhibiting Rf less than one indicating the *M. incognita* population was not sustained. A similar greenhouse experiment by Ibrahim et al. (1993) also revealed variation in host suitability among cultivars of barley, corn, oat, rye, sorghum, triticale, and wheat. However, in this greenhouse experiment, corn was more susceptible to *M. incognita* infection than the small grains tested except for barley. Barley was the most suitable host of *M. incognita* (Ibrahim et al., 1993). These findings from 20 years ago are similar to our experiment indicating potential variability in the nematode suppressive capabilities of different small grain cultivars.

*Meloidogyne incognita field seasons 2019–2020 and 2020–2021*: Despite variations in plant growth and grain yield across the small grains, nematode populations did not vary significantly. Total seasonal nematode numbers per gram of root were statistically similar between the cultivars within their respective small grain group. Observed differences in plant height and grain yield were likely due to attributes that were crop-specific rather than to *M. incognita* infection. In an experiment by Roberts et al. (1981), the effects of *M. incognita* on winter wheat grain yield and the influence of soil temperature and planting date on *M. incognita* development, reproduction, and winter survival determined that *M. incognita* is capable of infecting autumn-sown wheat plants and completing one generation during the winter season. This experiment saw comparable results to our findings in our field experiments where there was no significant difference in grain yield between infested and non-infested plots (Roberts et al., 1981). Furthermore, the experiment also demonstrated there were no visible differences in top growth, plant height, leaf color, and amount of tillering in November and December between young *M. incognita* infected and non-infected plants (Roberts et al., 1981). In our experiment, in the *M. incognita* field in both years, rye cultivars consistently exhibited the greatest plant height, followed by oat and triticale, while barley and wheat cultivars were shorter on average. Johnson et al. (1981) observed this same superiority in shoot growth of rye cover crops reporting four times more shoot growth in winter rye than spring oats that were grown in the fall and then winter killed. Rye is known to be the taller and quicker growing of the cereal crops, but it also the hardiest, being widely adapted across most climate zones (SARE, 2012). Oats are another widely adapted cover crop recognized for its tall, upright growth that reaches heights 1 meter and greater (SARE, 2012). Triticale is not only a tall crop but offers a large canopy cover and performs well in less optimal environments (Ayalew et al., 2018). The tall stature of some rye, oat, and triticale cultivars can form a canopy that blocks sunlight from reaching weeds, allowing these cereal crops to outcompete weeds and enhance overall crop performance (Teasdale and Mohler, 2000). Allelopathic compounds are also known to be present in oat and rye roots that can naturally inhibit weed growth (Shirley et al., 1998) and nematode reproduction (Halbrendt, 1996). An experiment by Timper (2017) suggests that in addition to seeing improvements in soil structure, moisture retention, and weed control, growers may benefit from lower populations of *M. incognita* following a high-residue rye winter cover crop. Biomass yield near harvest ranged widely among the small grain cultivars, with oat cultivars yielding the greatest biomass followed by rye, barley, triticale, and wheat, respectively. Raper et al. (2008) demonstrated that high-residue small grains integrated into a conservative cotton production system, can be rolled when mature prior to cotton planting to conserve soil moisture. Oats are known to provide quick, weed-suppressing biomass that can provide sufficient ground cover as a mulch before low-till or no-till crops (Shirley et al., 1998). Additionally, oat cultivars had greater grain yields compared to all other small grain cultivars, followed by triticale, barley, rye, and wheat, respectively. Oats can be harvested for grain but require a longer growing season in the Southeastern U.S. to optimize yield. In general, small grains planted too late are subject to winter damage that negatively affects yield (Mask et al., 2017). Wheat is widely grown in the winter as a cash grain in addition to its cover crop benefits, however, for wheat to be a successful grain crop it must be managed as such by selecting the right variety, timely planting, and monitoring soil fertility (SARE, 2012). Developmental variety, ‘OG9484’ wheat produced significantly greater grain yield compared to the other three wheat cultivars analyzed. It may be that the ‘OG9484’ cultivar is more adaptive to the Southeast region. These results highlight the intricate interplay between nematode management and agronomic performance in small grain cover crops. This knowledge is essential for developing tailored strategies that optimize both nematode control and crop productivity. Although there were no significant differences in total seasonal *M. incognita* egg population amongst the small grain cover crops, barley cultivars could potentially act as favorable hosts for *M. incognita* in the Southeast, suggesting a need for careful consideration when selecting cover crops based on specific nematode management objectives.

*Rotylenchulus reniformis field seasons 2019–2020 and 2020–2021*: The same superiority in shoot growth of rye, oat, and triticale cultivars, respectively, that was observed in the *M. incognita* field was also observed in the *R. reniformis* field. Barley and wheat cultivars, on average, were the shortest of the winter small grain crops, which was also consistent with observations from the *M. incognita* field. Barley cultivars, although not advantageous in shoot growth or biomass production in the *R. reniformis* field, outperformed all other small grain crops in grain yield. Barley cultivars, along with oat and rye, also had the highest total seasonal *R. reniformis* eggs per gram of root. Overall, grain yield was low in the *R. reniformis* field compared to results from the *M. incognita* field, which may be due to poor field conditions following unseasonable weather that was observed in the second trial year, which submerged the plots on several occasions. There was little variability in grain yield between cultivars within their respective crop groups, with two notable exceptions. ‘TAMO 411’ oat and ‘Abruzzi’ rye demonstrated significantly higher grain yields compared to other oat and rye cultivars tested. Oat cultivars overall were the top producers of biomass, which was also noted in the *M. incognita* field. These results further confirm the premise that nematode populations may not influence crop performance of winter small grains, but more importantly as suitable hosts, they may affect nematode levels that a subsequent cotton crop may be exposed to. Jones et al. (2006) determined in a cover crop field experiment utilizing wheat and rye in addition to leguminous cover crops that no increase in *R. reniformis* populations was observed over two consecutive cover cropping seasons under natural field conditions. However, when these cover crops were observed in rotation with cotton, there was a substantial increase in *R. reniformis* population density 120 days after emergence of cotton plants (Jones et al., 2006). In contrast, some studies show that cover crops like rye did not affect reniform nematode populations but did reduce cotton yields in certain regions (Molin and Stetina, 2013). More research is needed on the host suitability of winter small grain cover crops to *R. reniformis* to determine the best choice of a useful rotation crop in Southeastern cotton production.

*Forage quality 2019–2020 and 2020–2021*: The forage quality experiments provided valuable insights into the nutritional value of the small grains tested. Plant height trends were consistent with findings in the nematode field experiments, with rye, oat, and triticale cultivars being taller than barley and wheat. In terms of forage quality, plant height serves as an important indicator of forage biomass and potential grazing or hay yield (Sollenberger and Cherney, 1995). The fresh biomass weights collected in these experiments were collected during each crop’s vegetative stage when samples were required to be collected for forage quality analysis versus collecting fresh biomass weights near harvest in the nematode experiments. Rye cultivars showed superior biomass production during the vegetative stage. Nonetheless, in terms of forage production, the suitability of a cover crop to be utilized as a forage is reliant upon its forage quality (Snapp et al., 2005). Oat, barley, and rye cultivars had optimal CP concentrations for the fall and winter seasons (Ditsch and Bitzer, n.d.). Triticale and wheat cultivars averaged slightly outside of the optimal range, which indicates potential for these crops to cause digestive issues if CP concentrations become too high (National Research Council, 2000). The ADF and NDF concentrations for all small grains during the vegetative stage indicated potential for high fiber content and high digestibility values close to 30% and 40%, respectively (Rocateli and Zhang, n.d.) with averages ranging between 25% to 27 % for ADF and 43% to 48 % for NDF. These small grains offer a well-rounded nutritional profile, providing sufficient fiber, digestibility, and available energy measured by their TDN (Rodehutscord et al., 2016). While high TDN levels are generally desirable for meeting energy needs of grazing animals, it’s crucial to maintain a balanced approach. Excessively high TDN levels, without considering other nutritional factors, could potentially lead to digestive issues (Owens et al., 1998). As plants mature, their TDN levels tend to decrease, further highlighting the importance of proper forage management and timing to ensure optimal forage quality and animal health (Rocateli and Zhang, n.d.). Among the small grains, oats stand out as the most digestive-friendly option due to their high fiber content, minimizing the risk of gastrointestinal disturbances (Dhuyvetter, n.d.). Rye is generally less palatable compared to other small grains, while wheat should be consumed in moderation to reduce potential digestive issues (Dhuyvetter, n.d.). Our research has shown variations in fiber content among these small grains, with rye and triticale cultivars exhibiting the highest levels, followed by wheat and barley, while oat cultivars had the lowest fiber content. Barley and triticale forages are known for their superior nutrient profile and enhanced digestibility compared to oats and wheat (Khorasani et al., 1997). These differences in fiber and nutrient composition highlight the importance of carefully selecting and incorporating the appropriate small grains into livestock diets to ensure optimal digestive health and overall animal performance. Percent ADF is combined with percent NDF to produce the RFV index, which is used to compare forages to the standard forage quality found in full bloom alfalfa hay (Undersander et al., 2010). Alfalfa hay at full bloom has approximately 53 % ADF and 41 % NDF that establishes a baseline RFV of 100 (Moore and Undersander, 2002). This baseline is a popular metric used by both buyers and sellers to determine the best dollar value for quality hay. Forages possessing RFV indices above 100 are considered to be of superior quality. (Rocateli and Zhang, n.d.). All small grains sampled in the vegetative phase for forage analysis had RFV indices above 100, indicating superior quality compared to the alfalfa hay standard.

*Summary:* This comprehensive experiment provides crucial insights into the complex relationships between small grain cover crops, nematode populations, and agronomic performance. Through a combination of greenhouse and field experiments, this research revealed varying host suitability across the small grain cultivars for *M. incognita*, with some triticale and oat varieties demonstrating potential as poor hosts. Notably, field experiments for both *M. incognita* and *R. reniformis* indicated that nematode populations did not significantly impact crop performance, suggesting that crop-specific attributes play a more substantial role in determining plant height, biomass, and grain yield. Each small grain crop exhibited distinct advantages: Oats demonstrated versatility in nematode management and high yields; rye excelled in height and biomass production, potentially enhancing weed suppression (Teasdale and Mohler, 2000); triticale offered balanced performance; barley showed high grain yield potential, particularly in *R. reniformis*-infested fields; and wheat provided superior CP content for forage. Forage quality analysis overall revealed that all small grains offer superior nutritional value compared to the alfalfa hay standard, with each crop presenting unique nutritional profiles. These diverse attributes underscore the importance of selecting cover crops based on specific management goals, including nematode suppression, biomass production, forage quality, and overall soil health improvement.
